# Simultaneous purification of dihydrotanshinone, tanshinone I, cryptotanshinone, and tanshinone IIA from *Salvia miltiorrhiza* and their anti-inflammatory activities investigation

**DOI:** 10.1038/s41598-018-26828-0

**Published:** 2018-05-31

**Authors:** Hongwei Gao, Liting Huang, Fang Ding, Ke Yang, Yulin Feng, Hongzhen Tang, Qiong-ming Xu, Jianfang Feng, Shilin Yang

**Affiliations:** 1 Guangxi University of Chinese Medicine, Nanning, 530000 China; 2 State Key Laboratory of Innovative Drug and Efficient Energy-Saving Pharmaceutical Equipment, Jiangxi University of Traditional Chinese Medicine, Nanchang, 330004 China; 3 College of Pharmaceutical Science, Soochow University, Suzhou, 215123 China

## Abstract

Dihydrotanshinone, tanshinone I, cryptotanshinone, and tanshinone IIA are major lipid-soluble constituents isolated from *Salvia miltiorrhiza* Bunge (Danshen). In the present study, a systematic method was developed to simultaneously isolate and purify those compounds using macroporous adsorption resins and semi-preparative HPLC with a dynamic axial compress (DAC) system. The Danshen extract (95% alcohol) was divided into three fractions using different concentrations of alcohol (0%, 45%, and 90%) on D101 column. The content of total tanshinones of 90% alcohol eluent (TTS) was over 97%. Furthermore, the anti-inflammatory effects of those samples were investigated on LPS-stimulated RAW264.7 cells and three animal models. The results showed that the anti-inflammatory effect of TTS *in vitro* was superior to the one of any other sample including 0% and 45% eluent, and total tanshinones capsules. In addition, TTS exhibited a stronger anti-inflammatory effect than that of dihydrotanshinone, tanshinone IIA, cryptotanshinone, and tanshinone I, respectively. For animal models, TTS could significantly suppress xylene-induced ear oedema and rescue LPS-induced septic death and acute kidney injury in mice. In summary, the separation process developed in the study was high-efficiency, economic, and low-contamination, which was fit to industrial producing. TTS is a potential agent for the treatment of inflammatory diseases.

## Introduction

The inflammation is always triggered by damage to organisms, which plays a defensive role in injury or infection^1^. However, durable inflammation has more often than not lead to the inflammatory diseases, such as sepsis, endotoxemia, asthma and inflammatory bowel disease (IBD), *etc*.^2,3^. Specifically, sepsis is largely induced by the hyper-inflammatory responses, which involves the initiation and amplification of the innate immune system and cytokines release like TNF-α, IL-1β, IL-6 *etc*.^4^. As it stands now, there is no an effective drug with fewer side effects in the clinic to rescue septic death. Therefore, it is imperative to explore a more effective and less side effect drug for the treatment of inflammatory diseases.

Danshen, one of the most popular traditional Chinese medicines in Asian countries, has been used extensively for the treatment of cardiovascular diseases, cerebrovascular diseases, and various inflammatory diseases^5,6^. Furthermore, Danshen, as a dietary supplement, is the first traditional Chinese medicine that is documented in USP 37-NF32. Tanshinones are mainly lipophilic active constituents isolated from the root of Danshen. As it stands now, more than 40 tanshinones have been isolated and identified^7^. Of these tanshinones, dihydrotanshinone (DTAN), tanshinone I (TANI), cryptotanshinone (CTAN), and tanshinone IIA (TANA) are major diterpenes in Danshen^8,9^. Following the traditional application, the cardiovascular protective effect of tanshinones has been widely investigated^10^. The anti-cancer effect of tanshinones has drawn attentions of many researchers in recent years^7,8^. Specifically, the four compounds were always chosen as target representing Danshen to investigate its anti-inflammatory activity^11,12^. In China, total tanshinones capsules (TTC) that were just prepared by 95% alcohol extract were employed as an anti-inflammatory medicine in the market (Z13020110). However, the anti-inflammatory effect of TTC was not satisfactory due to its crude preparation. Therefore, the innovative separation and effective methods to prepare Danshen samples or obtain pure compounds from raw Danshen extract are indispensable for the research and development of Danshen products.

The conventional ways to separate tanshinones were performed on extraction, multi-step open column chromatography with silicon gels, and semi-preparative HPLC. However, these assays did not fit the bill of the large-scale industrial production due to the onerous work and great consumption of solvents. Recently, macroporous adsorption resins were widely used to separate and enrich diverse compounds in industrial production for their high efficiency and low-cost quality^13^. In addition, the advantages of macroporous adsorption resins (MARs), such as the ideal pore structure, unique adsorption properties, less solvent consumption, affable environmental management and easy regeneration make them priority for large-scale production industrially^14–16^. Therefore, it is the vital part to select optimal MARs for the enrichment of target constituents.

**Figure 1:**
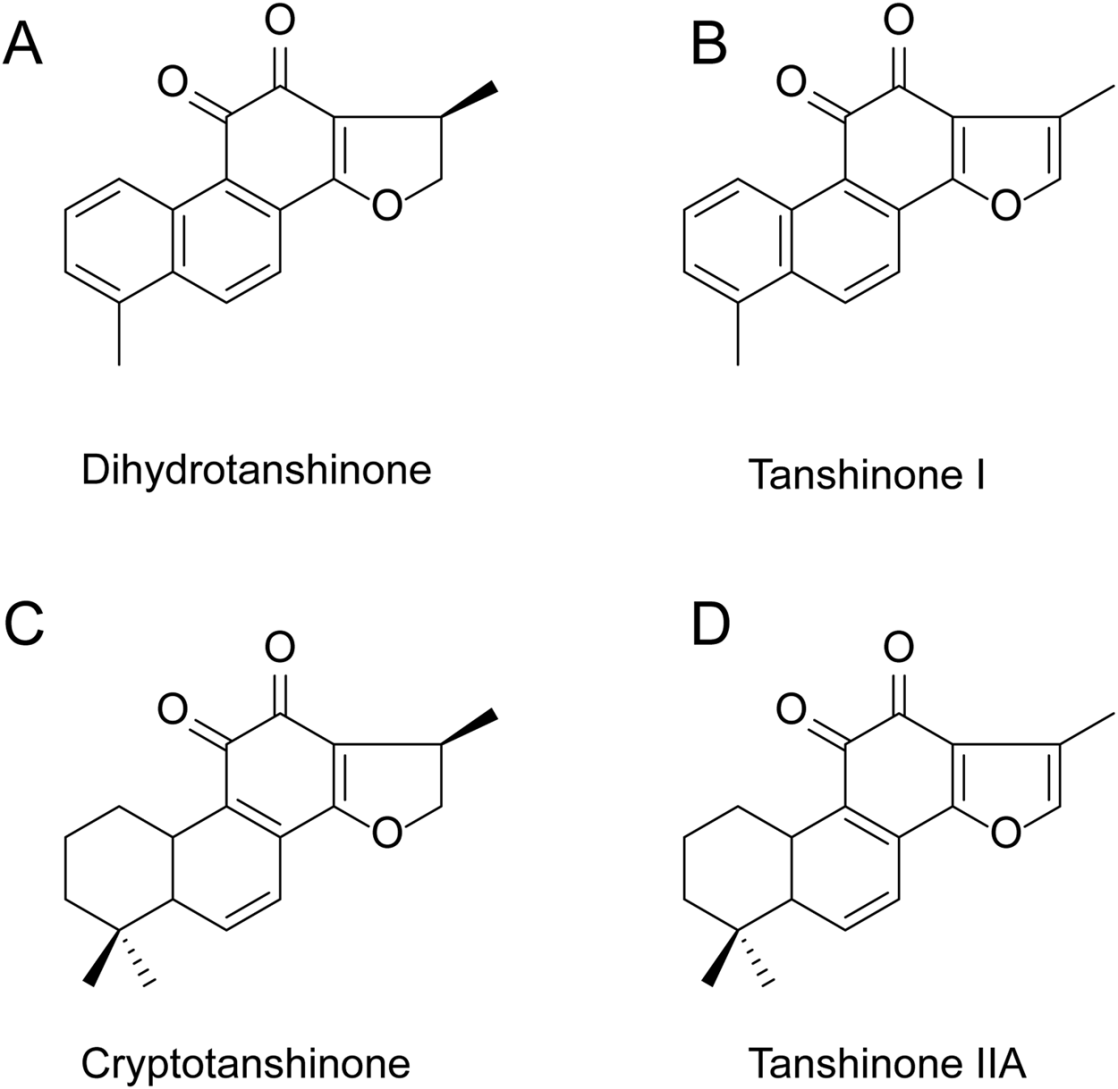
Chemical structures of pure tanshinones. (**A**) Dihydrotanshinone (DTAN). (**B**) Tanshinone I, (TANI). (**C**) Cryptotanshinone (CTAN). (**D**) Tanshinone IIA, (TANA).

The technology of dynamic axial compression (DAC) has been developed fast in industrial separation due to its stable performance, high efficiency, and good repeatability for the large-scale production. A deluge of compounds such as pulchinenoside B4 and B5, saikosaponins A, B, and C, zopiclone, *etc*. were separated and purified using DAC columns with reversed-phase ODS^17–19^. However, as it stands now, there are no related studies to explore for purifying tanshinones using DAC columns with reversed-phase ODS. In this study, an effective preparative method was developed to simultaneously isolate and purify DTAN, TANI, CTAN, and TANA (Fig. 1) using MARs and preparative reversed-phase HPLC with a DAC column system, which provides a novel method for large-scale purification of tanshinones from natural resources.

## Results and Discussion

### The static adsorption and desorption experiments

The adsorption capabilities of MARs are not only associated with the polarity or chemical structure of adsorbates, but also with the characteristics of adsorbents such as the unique surface area, pore diameter, pore volume and so on, and even with adsorption medium^13^. For the four tanshinones, non-polar resins are more applicable to adsorption of them. In this study, seven MARs (Table 1) were employed to determine the static adsorption and desorption capability. In terms of our experiments, the non-polar resin D101 and HPD100 exhibited higher adsorption than other resins (Table 2), indicating that the similar polarity with tanshinones and the smaller pore size of resins for adsorption were optimal. In accordance with their desorption capabilities (Table 2) and desorption ratios of the four tanshinones (Table 2), the seven resins were ranked as following: D101 ≈ HPD100 > HPD600 > XDA-6 > LX-11 ≈ LX-38 > AB-8. Therefore, D101 and PHD100 were selected in terms of their static adsorption and desorption to test their dynamic adsorption and desorption capacity.

**Table 1:** Physical characteristics of seven macroporous adsorption resins.

Resins	Polarity	Specific surface area	Pore size	Particular diameter
		(m^2^/g)	(nm)	(mm)
AB-8	weak	480~520	13.0~14.0	0.3~1.25
D101	nonpolar	650~700	8.5~9.0	0.3~1.25
HPD100	nonpolar	400~600	10.0~12.0	0.3~1.25
LX-11	nonpolar	≥460	30.1	0.3~1.25
LX-38	middle	≥550	26	0.3~1.25
XDA-6	middle	63	4.98	0.3~1.25
HPD600	polar	55~600	8	0.3~1.2

**Table 2:** Adsorption capacities, desorption capacities and ratios of seven resins.

Resins	Dihydrotanshinone	Tanshinone I	Cryptanshinone	Tanshinone IIA	
AB-8	23.0 ± 2.1	48.2 ± 1.0	55.0 ± 1.9	53.0 ± 1.9	Adsorption capacity (mg/g)
D101	28.2 ± 2.4	57.8 ± 2.0	57.5 ± 4.2	73.3 ± 3.9	
HPD100	29.1 ± 1.6	59.2 ± 2.2	57.5 ± 2.3	73.7 ± 2.8	
HPD600	26.5 ± 1.6	56.4 ± 2.9	55.9 ± 3.8	69.1 ± 4.4	
LX-11	27.7 ± 1.5	58.3 ± 1.3	52.3 ± 2.4	70.4 ± 3.5	
LX-38	27.3 ± 2.4	56.6 ± 2.4	66.9 ± 4.4	68.8 ± 5.4	
XDA-6	28.2 ± 1.3	59.6 ± 1.2	63.0 ± 1.2	72.0 ± 6.2	
AB-8	9.1 ± 0.6	14.7 ± 1.1	12.2 ± 1.0	39.1 ± 2.2	Desorption (mg/g)
D101	23.3 ± 1.3	41.0 ± 1.2	41.2 ± 2.1	71.3 ± 4.1	
HPD100	23.8 ± 1.4	39.9 ± 1.4	37.1 ± 2.1	72.1 ± 3.1	
HPD600	21.6 ± 1.5	35.4 ± 2.8	29.8 ± 1.1	63.0 ± 3.6	
LX-11	15.3 ± 1.1	25.5 ± 1.1	30.7 ± 1.2	60.4 ± 1.7	
LX-38	16.5 ± 1.8	26.8 ± 1.3	30.2 ± 1.2	59.9 ± 3.2	
XDA-6	21.4 ± 1.2	28.3 ± 1.5	32.5 ± 1.5	68.0 ± 1.8	
AB-8	39.57	30.5	22.18	73.77	Desorption ratio (%)
D101	82.62	70.93	71.65	97.27	
HPD100	81.79	67.4	64.52	97.83	
HPD600	81.51	62.77	53.31	91.17	
LX-11	55.23	43.74	58.7	85.8	
LX-38	60.44	47.35	45.14	87.06	
XDA-6	75.89	47.48	51.59	94.44	

### Dynamic separation on the two selected resins

The results of dynamic separation on the two selected resins were shown in Table S1. The adsorption capability of D101 for the four compounds was higher than that of HPD100. The target compounds could barely be detected by HPLC in the 30% ethanol elute and less 5% target compounds were detected in 45% eluent. The four compounds could be efficiently desorbed by 90% ethanol elute. Collectively, D101 was chosen for the enrichment of tanshinones. 45% and 90% ethanol were selected for the exclusion of other compounds and enrichment for the target compounds, respectively.

### Investigation of the percentage of ethanol solution for desorption process on D101

**Figure 2:**
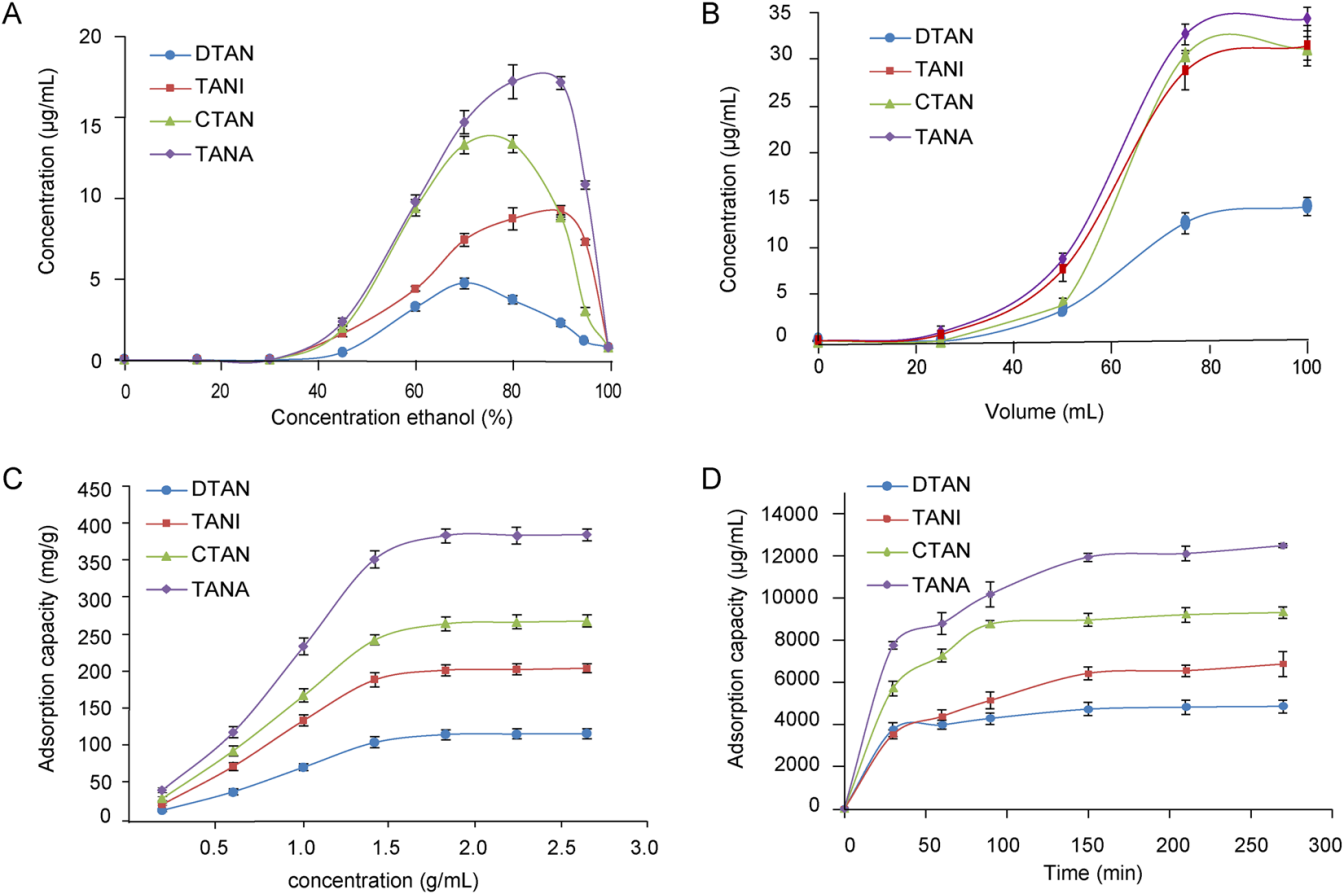
The adsorption and desorption behaviors of tanshinones on D101 resins. (**A**) Dynamic desorption curves. (**B**) Dynamic leakage curves. (**C**) Adsorption kinetic curves. (**D**) Adsorption isotherms.

After adsorption equilibrium at the optimal condition as aforementioned, the adsorbate-laden column was washed with deionized water (6 BV), and desorbed by gradient elution with 15%, 30%, 45%, 60%, 75%, 90%, and 100% ethanol (6 BV) at a flow rate of 4 BV/h. As shown in Fig. 2A, tanshinones could not be desorbed at 45% ethanol and could be completely desorbed at 90% ethanol. Therefore, the procedure of desorption was set as the following: deionized water, 45%, and 90% ethanol.

### Dynamic leakage curves on D101

The breakthrough point refers that the adsorbate concentration of eluent reaches 5% of inlet concentration. The results of leakage curve on D101 were obtained (Fig. 2B) in terms of the effluent volume and concentrations of target compounds. The breakthrough volume of target compounds on D101 was determined as 53 mL (1.4 BV).

### Adsorption kinetics on D101 resins

The adsorption kinetics curve on D101 was shown in Fig. 2C. The adsorption capacities of tanshinones on D101 resin increased with mounting adsorption time. The adsorption capacity had a growth spurt in the first 60 min, then slowed down and finally reached equilibrium after around 150 min for the target compounds, suggesting that D101 is slow adsorption resin for tanshinones. Therefore, the adsorption time was set to be longer than 150 min in the subsequent experiments.

### Adsorption isotherms on D101

Equilibrium adsorption isotherms on D101 were investigated at room temperature 25 °C using different concentrations of crude extract. During the dynamic adsorption, the feeding concentration and feeding volume were critical for the analytes loss. As shown in Fig. 2D, the adsorption capacity geared up but slowed down with the mounting feeding concentration of samples, and finally reached the adsorption saturation at 1.8 g/mL of crude extract. Therefore, the feeding concentration of crude extract was set as 1.8 g/mL.

### Large-scale enrichment on D101 resin

**Figure 3:**
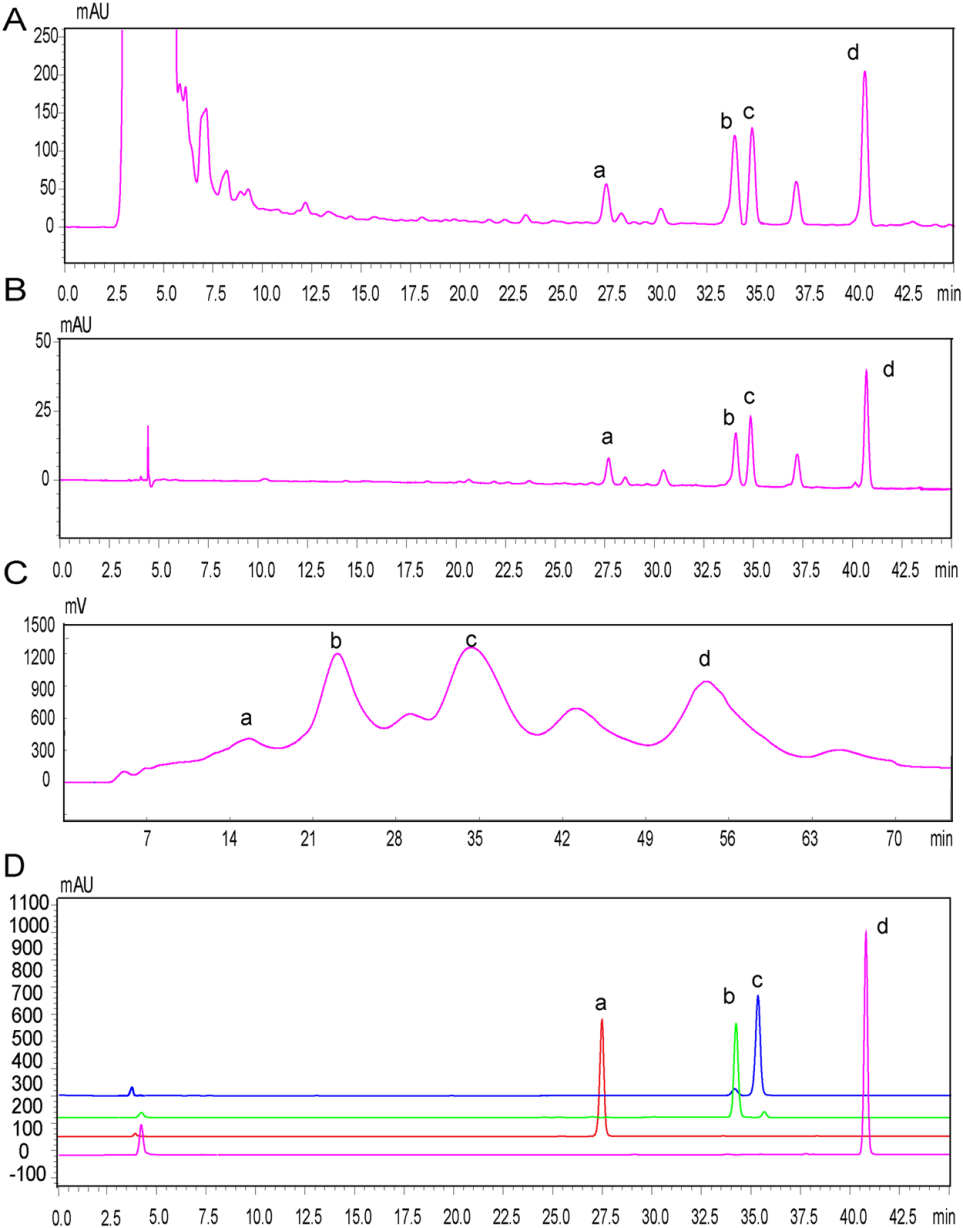
HPLC chromatograms. (**A**) HPLC chromatogram of 90% ethanol extract of raw materials extract. (**B**) HPLC chromatogram of 90% eluent sample (TTS). (**C**) DAC chromatogram of TTS. (**D**) HPLC chromatograms of pure tanshinones prepared by the semi-preparative HPLC-DAC system. a. DTAN, b. TANI, c. CTAN, d. TANA.

On the basis of optimal conditions aforementioned, like the crude extract 1.8 g/mL, the resin D101, the feeding sample volume 1.4 BV, the adsorption time 150 min, the large-scale enrichment of the target compounds was performed on enlarged glass column. The gradient elute solution was set as 0%, 45%, and 90%, respectively. The bed volume, the volume of elute solution, and the total feeding amount of raw Danshen extract (Fig. 3A) were enlarged by 120-fold. 90% eluent (TTS) were obtained which contained 5.87%, 15.62%, 30.23%, and 45.17% for DTAN, TANI, CTAN, and TANA, respectively (Fig. 3B).

### Preparative separation of DTAN, TANI, CTAN, and TANA using reversed-phase HPLC with ODC system

In the process of preparative purification, the different elution procedures were applied to obtain DTAN, TANI, CTAN, and TANA (Fig. 3C). Finally, the eluent procedure was selected as isocratic elution with 80% method at the flow rate of 200 mL/min. Through the whole purification procedure, DTAN, TANI, CTAN, and TANA were obtained with the purity of 96.2%, 97.1%, 96.3%, and 98.6%, respectively (Fig. 3D).

### TTS inhibited LPS-induced nitrite and NO in RAW264.7 cells

**Figure 4:**
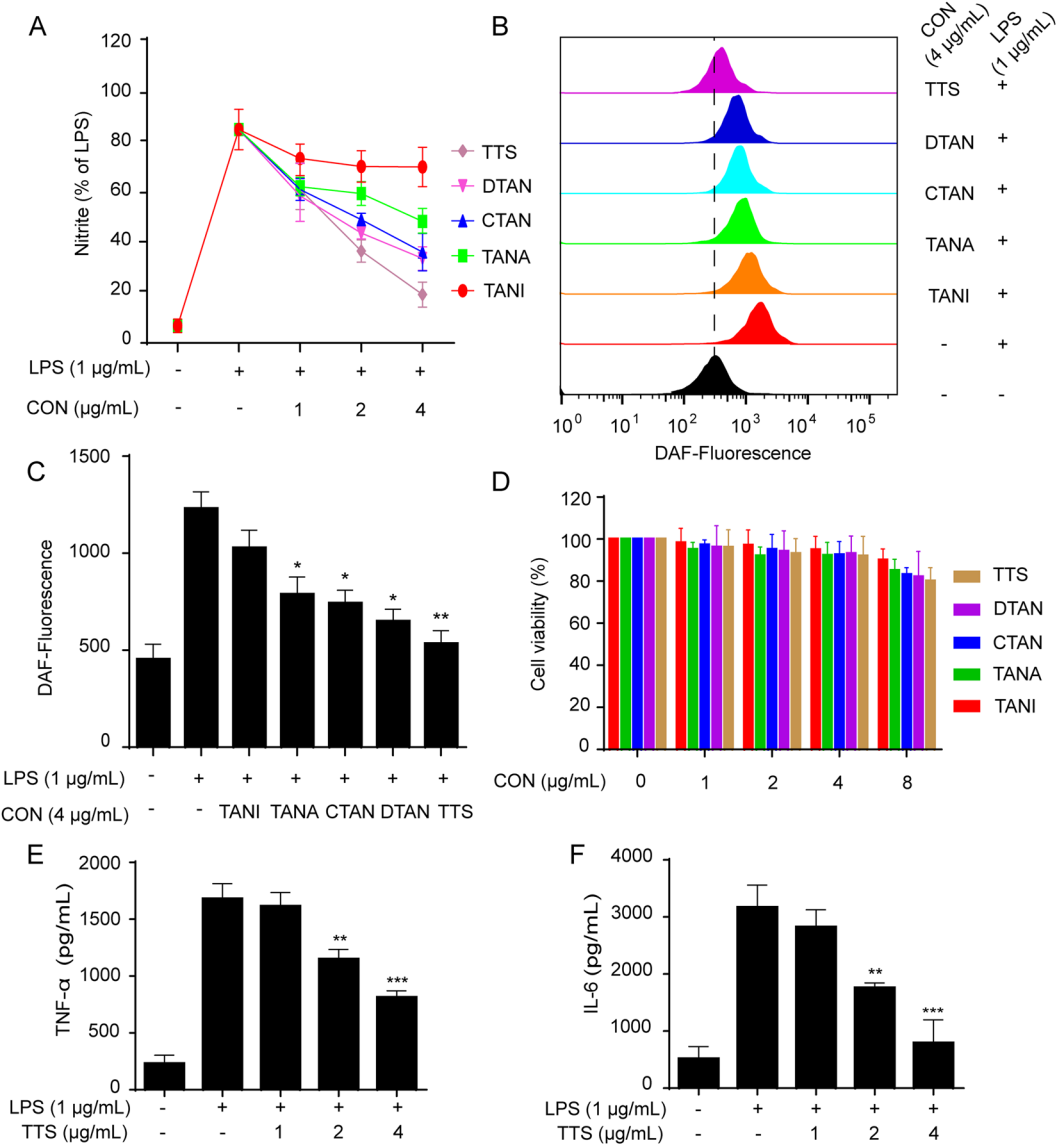
The inhibitory nitrite level and nitric oxide (NO), TNF-α, and IL-6 release of TTS in LPS-stimulated RAW264.7 cells. (**A**) Cells were pretreated with DTAN, TANI, CTAN, TANA, and TTS for 1 h before LPS (1 μg/mL) co-culture for 24 h. The nitrite levels of those samples were determined by Griess assay. (**B**) Cells were individually treated with DTAN, TANI, CTAN, TANA, and TTS for 6 h and then labeled with DAF-FM (1 μM) for another 1 h. The NO release was determined by the flow cytometry. (**C**) The quantitative fluorescence intensity was statistically determined. (**D**) Cells were treated with DTAN, TANI, CTAN, TANA, and TTS respectively for 24 h. The cell viability was investigated by MTT assay. (**E** and **F**). Cells were pretreated with TTS for 1 h and then stimulated with/without LPS (1 μg/mL) for 16 h. The ELISA assay was employed to detect levels of TNF-α and IL-6 in the culture medium. ^*^p < 0.05, ^**^p < 0.01, and ^***^p < 0.01 versus LPS-treated group.

Synthesized endogenously from L-arginine by nitric oxide synthases (NOSs), NO becomes an inflammometer to modulate important cellular signaling involved in immunity and inflammation^20^. LPS-induced NO production in RAW264.7 macrophages has been considered as the convenient and credible method for anti-inflammation screening^21^. The Griess assay for the determination of nitrite resulted from the reaction of NO with O_2_ was used as an effective and efficient method to quantitate NO production^22,23^. Therefore, these compounds were primarily screened with an LPS-stimulated RAW264.7 macrophage cell model for their anti-inflammatory activities. As shown in the results, TTS exhibited significant inhibitory effect on nitrite production and NO level, (Fig. S1). The inhibition effect on nitrite level and NO release of 0% and 45% alcohol eluent and total tanshinones capsules (TTC) was inferior to that of TTS. In addition, the effect of TTS *in vitro* was superior to the one of any other sample, sequentially followed by DTAN, CTAN, TANA, and TANI (Fig. 4A,B,C), which suggested that there should be drug-drug interactions and possibly be synergistic effect among them. Furthermore, TTS significantly suppressed LPS-induced cytokines release like TNF-α and IL-6 in RAW264.7 cells (Fig. 4E,F). Combination of nitrite level and NO, TNF-α, and IL-6 release assays, we speculated that TTS exhibited a significant anti-inflammatory effect on LPS-stimulated RAW264.7 cells. Therefore, TTC is needed to be further prepared for better anti-inflammatory activity. TTS was chosen for further study *in vivo*. In addition, MTT results demonstrated that samples with indicated concentrations exhibited no cytotoxicity (Figs 4D and S1).

### TTS suppressed xylene-induced ear oedema

**Figure 5:**
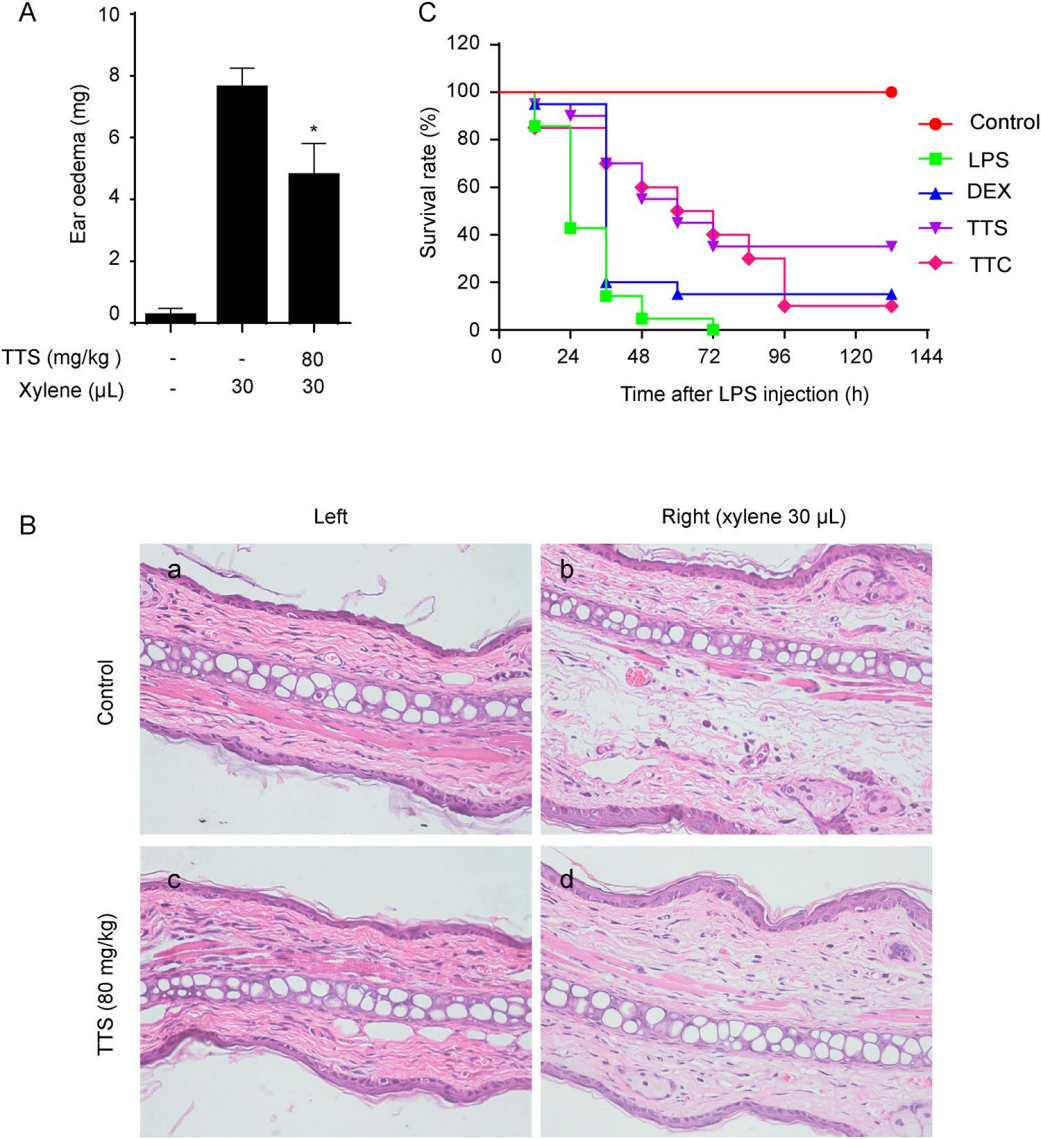
TTS suppressed xylene-induced ear oedema and rescued LPS-induced septic shock in mice. (**A**) Ear oedema was established by xylene injection. Pretreatment with TTS (80 mg/kg) for 2 h before xylene injection. One hour later, ear weight was measured following the Methods. Values are shown mean ± SD (n = 10). (**B**) Histopathological examination of the ear sections. a. The left ear of mice not treated with ISO was regarded as a vehicle control. b. The right ear of mice treated with xylene (30 μL) but not with TTS was regarded as the model control. c. The left ear of a mouse pretreated with TTS (80 mg/kg, i.p.) for 2 h but not treated with xylene. d. The right ear of a mouse pretreated with TTS (80 mg/kg, i.p.) for 2 h and then treated with xylene (30 μL) for another 1 h. The ear tissue samples were stained with H&E as described in the Methods section. (H&E, original magnification, 400×). (**C**) 20 mice per group pretreated with vehicle or TTS (80 mg/kg, i.p.) for 2 h before LPS (20 mg/kg, i.p.) injection. DEX (5 mg/kg, i.v.) was used as positive control. Survival rates of these mice were observed for next 132 h. ^*^P < 0.05 compared to xylene alone group.

Xylene is always employed to induce an acute inflammatory response in animal models, which results in ear oedema when administrated to the surfaces of the ear^24^. In terms of the advantages of xylene-induced ear oedema in mice, the anti-inflammatory model is always employed to evaluate the antiphlogistic effect for drug screenings of TTS^24^. In our study, TTS significantly inhibited xylene-induced ear oedema (Fig. 5A). In addition, the results of ear sections H&E staining indicated that TTS decreased xylene-induced oedema, cell necrosis, and lymphocytic infiltration (Fig. 5B).

### TTS rescued LPS-induced septic death

**Figure 6:**
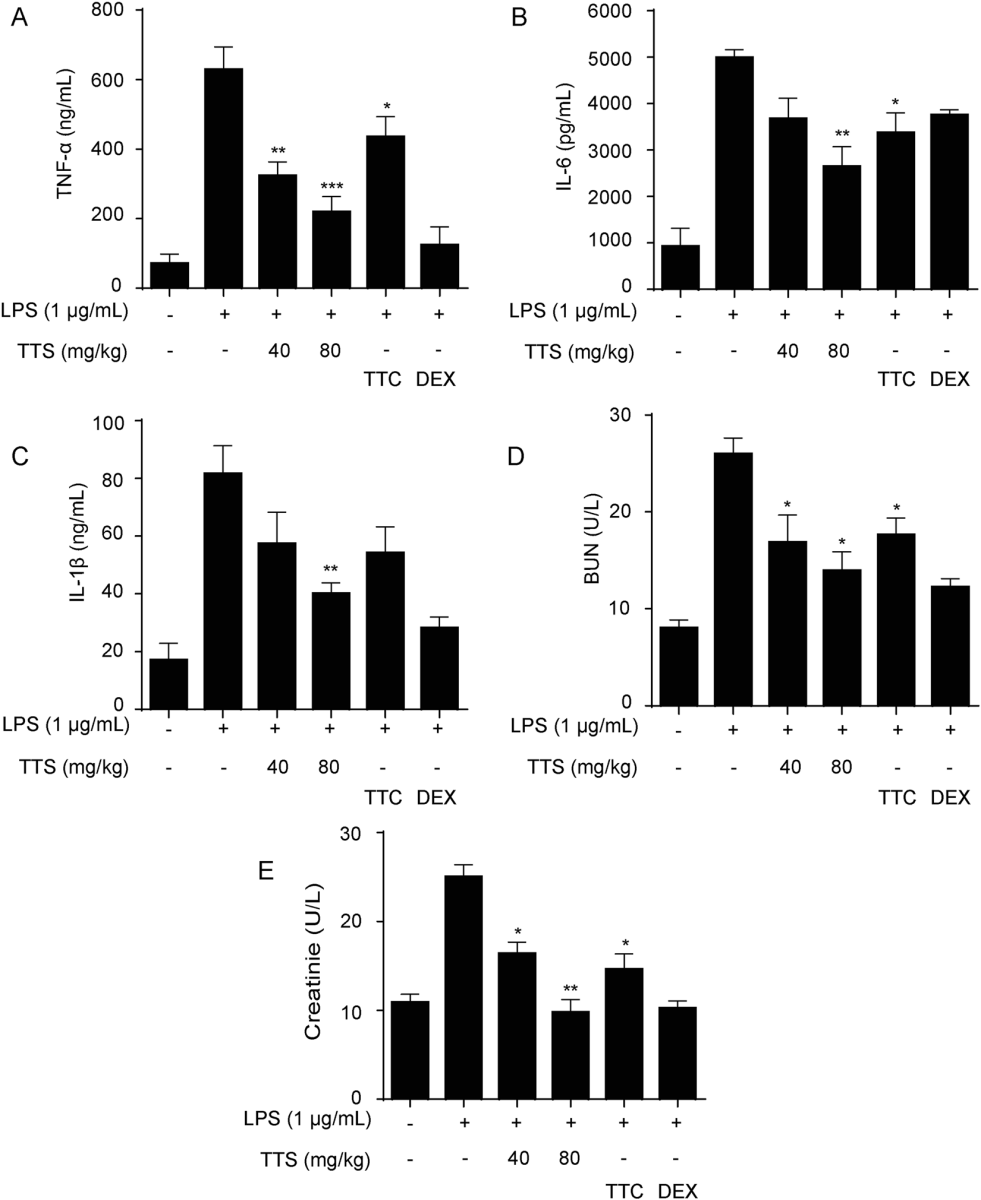
TTS suppressed inflammatory cytokines release and renal injury makers in BALB/C mice. Mice were pretreated with TTS (40 and 80 mg/kg, i.p.), TTC (80 mg/kg) for 2 h before LPS (10 mg/kg, i.p.) injection. After 12 h, the blood samples were collected *via* retro-orbital route under anesthesia. The inflammatory cytokines like TNF-α (**A**), IL-6 (**B**) and IL-1β (**C**) and creatinine (**D**) and blood urea nitrogen (**E**) in serum were determined by ELISA kits. DEX (5 mg/kg, i.v.) was used as positive control. ^*^P < 0.05, ^**^P < 0.01, ^***^P < 0.001, compared to LPS alone group (n = 10).

Sepsis, a life-threatening syndrome caused by inflammatory cascades following the invasion of bacteria, viruses, etc. and release of various toxic products like LPS, leads to fever, tachypnea, high white blood cells, multiple organ dysfunction syndromes, and even death^25,26^. Accompanying with the severe septic syndrome, several inflammatory cytokines like TNF-α, IL-1 and IL-6 have been involved in the pathogenesis of septic shock^26^. In addition to those inflammatory mediators, an overproduction of NO was demonstrated to play an important role in LPS-stimulated models^27,28^. Thus, whether inhibition of over release of those inflammatory cytokines or not is an important indicator treatment with the septic syndrome. As shown in Fig. 5C, to the LPS-induced septic model, all mice died within 72 h of LPS injection. However, 1 h pretreatment with TTS (80 mg/kg) could significantly rescue LPS-induced septic death. The survival rate was around 40% at 132 h, which was more effective than that of DEX and TTC. Besides, TTS significantly decreased the release of TNF-α, IL-1β, and IL-6, whose treatment effect was better than that of TTC (Fig. 6A,B,C).

### TTS reversed LPS-induced acute kidney injury

**Figure 7:**
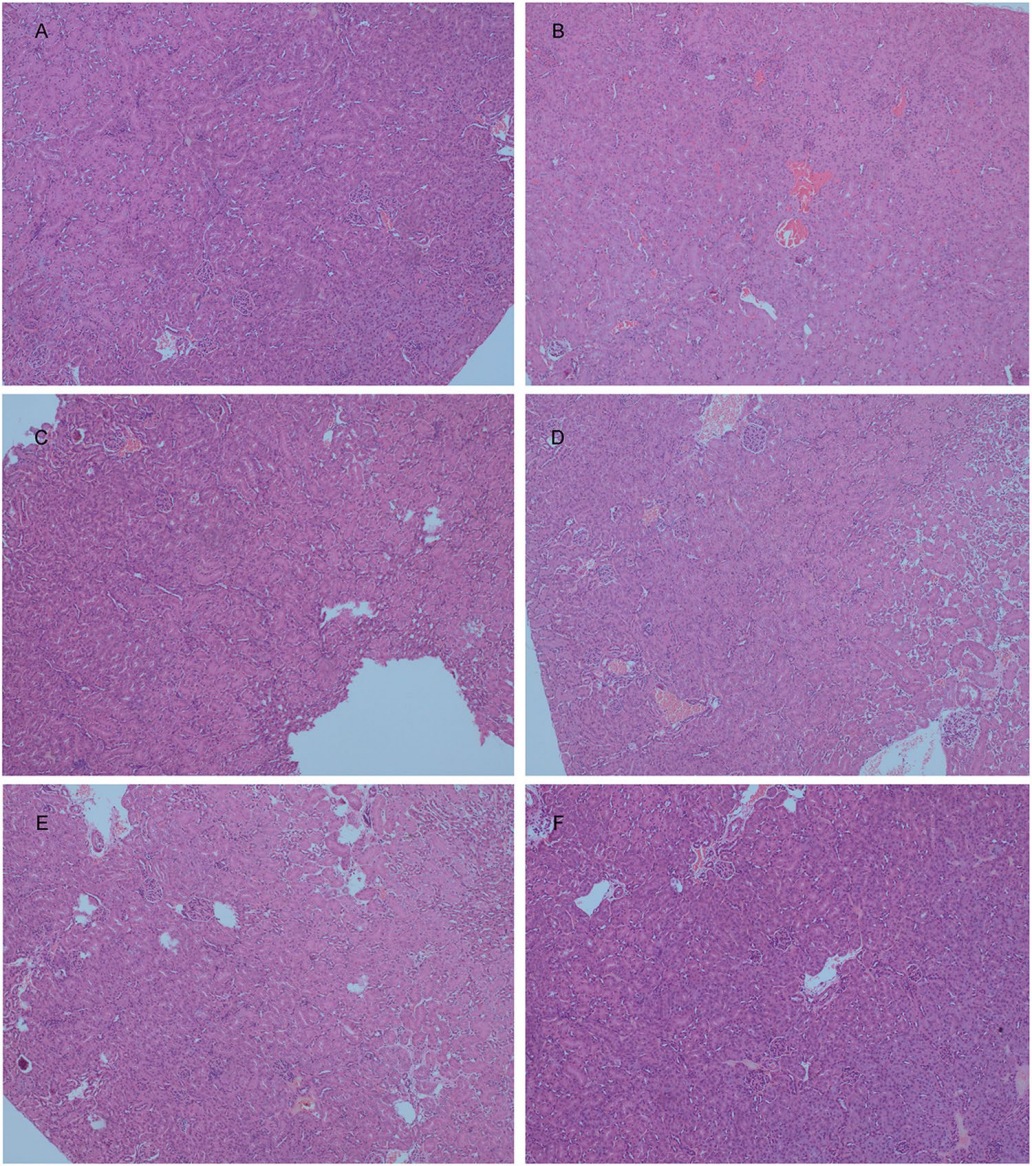
Histopathological examination of kidney tissue of LPS-stimulated mice treated TTS (H&E, original magnification, 100×). The kidney tissue was collected from the mice that were used to detect inflammatory cytokines. (**A**) Normal group; (**B**) LPS (10 mg/kg, i.p.) group; (**C**) TTC (80 mg/kg, i.p.) group; (**D**) TTS (40 mg/kg, i.p.) group; (**E**) TTS (80 mg/kg, i.p.) group; (**F**) Positive control group, DEX (5 mg/kg, i.v.).

Acute kidney injury (AKI) is an abrupt or rapid loss of kidney function^29,30^, leading to an increase of serum creatinine and urine level due to the reduced urine output. AKI is a worldwide healthy problem and there is no effective drug available for its treatment. LPS has been identified as one of the most important factors that lead to AKI^31^. LPS could upregulate the production of pro-inflammatory cytokines TNF-α, IL-6, and IL-1β, which lead to the development of AKI^32,33^. In this study, TTS sharply rescued mice kidney function and decreased LPS-induced urine and creatinine level (Fig. 6D,E). In the control group, the H&E staining displayed normal kidney tubules (Fig. 7A). In LPS-challenged mice, the renal lesions marked the oedema of renal tubular epithelial cells, renal interstitial oedema of epithelial cells, and leukocyte infiltration were significantly observed (Fig. 7B). TTS pretreatment significantly attenuated LPS-induced epithelial atrophy and necrosis and interstitial oedemas (Fig. 7C,D,E). DEX was used as positive control (Fig. 7F).

In summary, a method for simultaneous purification of DTAN, TANI, CTAN, and TANA from *Salvia miltiorrhiza* Bunge was established. The results of static adsorption/desorption and dynamic separating experiments indicated that D101 resin was superior to other six resins investigated for separating tanshinones. Further static and dynamic desorption/desorption experiments on D101 were performed to obtained optimal parameters. The further process was developed by preparative reversed-phase HPLC with a DAC column to obtain pure dihyrotanshinone, tanshinone I, cryptanshinone, and tanshinone IIA. In terms of these results, the established method was highly efficient, relatively economic, and environmentally protective, which exhibited good potential for large-scale production of these compounds for functional food and pharmaceutical application. Furthermore, TTS exhibited a significant anti-inflammatory activity *in vitro* and *in vivo*, which was superior to DTAN, CTAN, TANA, TANI, and TTC, respectively. Specifically, for three animal models, TTS significantly demonstrated to suppress xylene-induced mice ear oedema, rescue LPS-induced septic death, and reverse LPS-induced AKI. Therefore, TTS prepared from 95% alcohol extract of raw Danshen displays giant value for further research as an anti-inflammatory agent to substitute TTC in the market.

## Materials and Methods

### Materials

DTAN (>98%), TANI (>98%), CTAN (>98%), and TANA (>98%) purchased from Shun Bo Biological Engineering Technology Co., Ltd. (Shanghai, China) were determined by HPLC. Tanshinones capsules (TTC) were purchased from Hebei Xinglong Xili pharmaceutical co. Ltd. (Hebei, China). LPS (*Escherichia coli*, serotype 0111:B4), Griess reagent, Dulbecco’s modified Eagle’s medium (DMEM) and fetal bovine serum (FBS) were purchased from Life Technologies/Gibco Laboratories (Grand Island, NY, USA). ELISA kits for IL-6, IL-1β, and TNF-α were purchased from Neobioscience (Shenzhen, China). Propitious amounts of standards were dissolved in methanol to be used as stock solutions at the concentrations of 2.34 mg/mL for DTAN, 2.43 mg/mL for TANI, 3.37 mg/mL for CTAN and 3.67 mg/mL for TANA, respectively. Ethanol (analytical grade) and methanol (HPLC grade) were purchased from Shanghai Chemical Reagents Ltd. (Shanghai, China).

### Adsorbents

D101, HPD100, HPD600, LX-11, LX-38, and XDA-6 were purchased from Cangzhou Bon Adsorbent Technology Co., Ltd (Hebei, China). AB-8 was obtained from Tianjin Nankai Hecheng S&T Co. Ltd (Tianjin, China). The specifications of these MARs were summarized in Table 1. Prior to the adsorption experiments, the resins were weighted and pretreated by soaking in 95% ethanol for 24 h and then washed with distilled water to complete removal of ethanol. Subsequently, the resins were doused in 4% NaOH (m/m) for 6 h, washed with distilled water until the pH of the filtrate was around 7, soaked in 4% HCl (v/v) for 6 h, and washed with distilled until the pH of the filtrate was 7 in sequence. The treated resins (the wet weight 1.0 g) were dried to constant weight at 70 °C in blasting drying oven.

### Preparation of crude extract of Danshen

The roots of Danshen were collected from Bozhou, Anhui Province, China. Its botanical origin was authenticated by Prof. Xiaoran Li in Soochow University (Suzhou, China), where the voucher specimen was deposited. The roots (1 kg) were powdered, and then refluxed with 95% ethanol at the ratio 1:10 (sample and solvent ratio, w/v) for 2 h, repeating two times. The extracted liquids were pooled, filtered, and then concentrated by rotary evaporator under vacuum to remove completely the ethanol solvent. The crude extract was then diluted with deionized water at the ratio of raw material and extract to be 1.0 g/mL.

### HPLC analysis

HPLC analysis was performed on Shimadzu Prominence LC-20A liquid chromatographic system (Shimadzu instruments company, Tokyo, Japan) equipped with binary pumps, a PDA detector, and LC solution software. The Waters RP-C18 column (150 × 4.6 mm i.d., 5 μm, Waters Co.Milford.MA, USA) was employed at a column temperature of 30 °C. The mobile phase consisted of methanol (A) and water (B, with 0.1% acetic acid) with a flow of 1 mL/min using the subsequent gradient elution: 0–45 min, 55–90% A. The injection volume was 20 μL and the absorbance wavelength was selected at 254 nm.

### Static adsorption and desorption ratio resins screening

All MARs were screened through static adsorption/desorption ratio experiments, which were performed as follows: five aliquots of 25 mL sample solutions prepared as the aforementioned (one-fold dilution of stock solution) were put into 100 mL flasks comprised the same amounts of various hydrated resins (equal to 1.0 g dry resins). The tightly sealed flasks were shaken (160 rpm) for 6 h at 25 °C to reach adsorption equilibrium. The solutions were filtrated through 0.45 μm minipore filter and detected by HPLC. Subsequently, the resins were washed with deionized water for 3 times and desorbed with 30 mL 95% ethanol. Then the flasks were shaken (160 rpm) for 6 h at 25 °C. The desorbed solutions were analyzed by HPLC. The experiments were repeated in three times. The candidate resins were screened by their properties of absorption/desorption ratios^34^. The subsequent equations were used to quantify the capacities of adsorption and desorption as well as the ratios of desorption.

Adsorption evaluation:

1 $${{\rm{Q}}}_{{\rm{e}}}=({{\rm{C}}}_{0}-{{\rm{C}}}_{{\rm{e}}}){{\rm{V}}}_{i}/{\rm{W}}$$

where Q_e_ indicates the adsorption capacity at adsorption equilibrium (mg/g dry resin); C_0_ and C_e_ are the initial and equilibrium concentrations of solutes in the solutions, respectively (mg/mL); V_*i*_ is the volume of the initial sample solution (mL); W is the dry weight of the tested resins (g).

Desorption evaluation:

2 $${{\rm{Q}}}_{{\rm{d}}}={{\rm{C}}}_{{\rm{d}}}{{\rm{V}}}_{{\rm{d}}}/{\rm{W}}$$

3 $${\rm{D}}={{\rm{C}}}_{{\rm{d}}}{{\rm{V}}}_{{\rm{d}}}/({{\rm{C}}}_{0}-{{\rm{C}}}_{{\rm{e}}}){{\rm{V}}}_{i}\times 100{\rm{ \% }}$$

where Q_d_ is the desorption capacity after adsorption equilibrium (mg/g dry resin); C_d_ is the concentration of solutes in the desorption solution (mg/mL); V_d_ is the volume of the desorption solution (mL); D is the desorption ratio (%); C_0_, C_e_, V_*i*_, and W are the same as formula (1).

### Dynamic adsorption/desorption tests on the selected resins

Dynamic adsorption and desorption experiments were performed on the open glass columns (30 × 2.0 cm i.d.) loaded with pretreated hydrated selected resins (D101 and HPD100). The 5 mL stock solutions (the concentration was 1 g/mL) prepared in section 2.3 were loaded on the various columns. Subsequently, the static adsorption in the glass columns was overnight to reach the adsorption equilibrium. For the elution process, the deionized water, 15%, 30%, 45%, 60%, 70%, 80%, 90%, and 100% were employed sequentially to load into the columns for 6 bed volumes (BV) with eluent rate of 2.0 mL/min. The eluents were condensed by rotary evaporator and detected by HPLC. To investigate dynamic leakage curves experiments, the prepared samples were through the column loaded D101 at the flow rate of 2.0 mL/min. After finishing loading sample, turn off the column till complete adsorption equilibrium. The concentration of the four compounds was detected by HPLC.

### Adsorption kinetics on D101

The adsorption kinetics curves of DTAN, TANI, CTAN, and TANA on the selected D101 resin were further researched as the following process: adding 30 mL sample solution prepared as 2.3 (one-fold dilution of stock solution) into a 100 mL flask comprised the same amount of selected hydrated resin (equal to 0.2 g dry resin)^35^. The tightly sealed flask was shaken (160 rpm) for 12 h at 25 °C. Then the concentrations of DTAN, TANI, CTAN, and TANA in the adsorption solution was analyzed by HPLC at certain time intervals (30, 60, 90, 150, 210, 270, and 330 min).

### Adsorption isotherms on D101

The adsorption isotherms of DTAN, TANI, CTAN, and TANA on the selected resin were investigated. The assay used was previously reported^36^. Briefly, adding various concentrations of solutions to a 100 mL flask with the same amounts of hydrated resins (equal to 0.2 g dry resin). The tightly sealed flasks were shaken (160 rpm) for 12 h at 25 °C. The adsorption solutions were analyzed by HPLC. The concentration of the loaded sample was from 0.2 g/ml to 2.6 g/mL.

### Scale-up enrichment of DTAN, TANI, CTAN, and TANA from crude extracts of Danshen

Scale-up separation was conducted by around 200 fold as that of lab conditions. D101 resin (1500 g, dry weight) was packed in a glass column with a Bed Volume (BV) of 20.0 L (150 × 10 cm i.d.). Then 5.0 L of aqueous sample solution was subjected to the column. After sample loading and adsorption equilibrium, desorption was performed smoothly with 7 BV of water, 6 BV of 45% ethanol, and 6 BV of 90% ethanol at a flow rate of 6.5 L/h. The 90% ethanol effluent was dried with rotary evaporation machine. The last samples were also analyzed by HPLC to determine the contents and the recoveries of the four target compounds.

### Preparative separation of DTAN, TANI, CTAN, and TANA by reversed-phase HPLC with DAC column

The preparative HPLC was performed on a DAC column system (150 × 10 cm i.d., HB-DAC-100, Jiangsu hanbang science and technology company, China) filled with Duke ODS gel (10 μm, 1.8 kg). The column was extensively flushed with pure methanol before use. The process of preparative separation was conducted as follows: The DAC column was pre-equilibrated with 70% methanol. The 90% ethanol sample was dissolved in methanol (150 mL), then filtered with 0.45 μm micro membrane filter, and then loaded to the column at a flow rate of 100 mL/min. The column was washed with 80% methanol (60 min) to get DTAN, TANI, CTAN, and TANA in sequence. The eluting experiments were conducted at a flow rate of 300 mL/min and monitored at a UV wavelength of 254 nm at room temperature. The indicated peaks were collected and analyzed by HPLC to determine the contents and the recoveries of target compounds.

### Cell culture

RAW264.7 macrophages purchased from Cell Bank of the Chinese Academy of Sciences (Shanghai, China) were cultured in DMEM with 10% FBS. Cells were maintained at 37 °C under a humidified atmosphere of 5% CO_2_ in an incubator.

### MTT assay

RAW264.7 cells were planted into 96-well plate at a density of 10^5^ cells/well. Subsequently, 0% and 45% alcohol eluent, TTS, and TTC (1, 2, 4, and 8 μg/mL) were employed for co-culture with cells for 24 h, and the cytotoxicity was determined using MTT assay as previously reported^6^.

### Determination of nitrite and NO

RAW264.7 cells were cultured at a density of 5 × 10^5^/well in 24-well plates overnight. After pretreatment with indicated compounds (1, 2, and 4 μg/mL) for 1 h, the cells were co-cultured with LPS (1 μg/mL) for 24 or 6 h. The nitrite in culture media treatment for 24 h was investigated by Griess reagent. The nitric oxide (NO) of cells treated with 6 h were stained by DAF-FM diacetate and determined by the flow cytometry (Becto-Dickinson, Oxford, UK) using the FITC channel.

### ELISA assay

RAW264.7 cells were cultured at a density of 5 × 10^5^/well in 24-well plates overnight. Cells were pretreated with TTS for 1 h and then stimulated with/without LPS (1 μg/mL) for 16 h. According to manufacturer’s instruction, the ELISA assay was employed to detect levels of TNF-α and IL-6 in the culture medium.

#### Animal experiments

BALB/c mice (male, 6–8 weeks old, 18–22 g) were obtained from the Experimental Animal Center of Soochow University. All mice were reared in plastic cages with food and water under standard conditions (SPF) and air filtration (22 ± 2 °C, 12 h light/dark cycles). The study was in accordance with the Local Guide for the Care and Use of Laboratory Animals of Soochow University and was approved by the university’s Ethics Committee of Experimental Animal Center of Soochow University (No. IACUC2016-21).

#### Xylene-induced mice ear oedema

For xylene-induced mice ear oedema model^37^, BALB/C mice were administered with TTS (80 mg/kg, i.p.). 12 h later, 30 μL xylene was applied to the posterior and anterior surfaces of the right ear of mice. The left ear was viewed as a control group. One hour later, mice were euthanized and two ear punches (7 mm, i.d.) were collected and weighted. The indicator of the edema was presented by the increase in the weight of right ear punch comparing with the left ear.

#### Septic shock in mice

For septic shock model^38^, BALB/C mice were obtained from Experimental Animal Center of Soochow University. Sepsis was performed in mice by i.p. injection of LPS (20 mg/kg), TTC (80 mg/kg, i.p.) and TTS (80 mg/kg, i.p.) was pretreated before LPS injection. The survival rate of mice was investigated in 7 days. The study was performed in accordance with the Local Guide for the Care and Use of Laboratory Animals of the Soochow University.

#### Cytokines and renal injury makers in BALB/C mice

For the AKI model^29^, Mice were randomly divided into six groups: control group (saline, i.p.), TTS (40 and 80 mg/kg, i.p.), TTC (80 mg/kg, i.p.), LPS group (10 mg/kg, i.p.) and positive control dexamethasone (i.v.). 2 h before LPS injection, TTS (40 and 80 mg/kg, i.p.) and TTC (80 mg/kg, i.p.) were administered mice. After 12 h, blood samples were collected *via* retro-orbital route under anesthesia and cytokines (TNF-α, IL-6, and IL-1β) were examined by mouse ELISA kits. The levels of blood urea nitrogen and creatinine in serum were determined by Roche Modular P800 (Roche, Shanghai, China). Their kidneys were collected for further research.

#### Histopathological analysis

The ear or kidney tissues harvested were fixed in 10% formaldehyde. Then, the tissues were dehydrated in a series of alcohol, embedded in paraffin, and sliced. The sections were stained with hematoxylin and eosin (H&E) stain. The pathological changes of ear or kidney tissues were observed under a light microscope.

### Data analysis

All results were presented as means ± SD. For statistical analysis, the significance of the intergroup differences was analyzed with one-way ANOVA using GraphPad Prism 6.0 software. Statistically significant difference was defined as ^*^p < 0.05.

### Data availability

All the detailed data and materials are available from the corresponding authors Yulin Feng or Hongzhen Tang.

## Electronic supplementary material

Supplementary Information
