# Supplementary material for: Simultaneous purification of dihydrotanshinone, tanshinone I, cryptotanshinone, and tanshinone IIA from Salvia miltiorrhiza and their anti-inflammatory activities investigation
Source: Sci Rep. 2018 May 31;8:8460. doi: 10.1038/s41598-018-26828-0 (PMC5981213; doi:10.1038/s41598-018-26828-0)
Supplement: Supplementary file 1 — Supplementary Information [file 41598_2018_26828_MOESM1_ESM.pdf]

Simultaneous purification of dihydrotanshinone, tanshinone I, cryptotanshinone, and tanshinone IIA from *Salvia miltiorrhiza* and their anti-inflammatory activities investigation

Hongwei Gao[1], Liting Huang[1], Fang Ding[1], Ke Yang[1], Yulin Feng[2*], Hongzhen Tang[1*], Qiong-ming Xu[3], Jianfang Feng[1], Shilin Yang[1,2]

[1]Guangxi University of Chinese Medicine, Nanning 530000, China. [2]State Key Laboratory of Innovative Drug and Efficient Energy-Saving Pharmaceutical Equipment, Jiangxi University of Traditional Chinese Medicine, Nanchang 330004, China. [3]College of Pharmaceutical Science, Soochow University, Suzhou 215123, China. Correspondence and requests for materials should be addressed to Y. Feng (fengyulin2003@126.com) or H. Tang (383744825@qq.com).

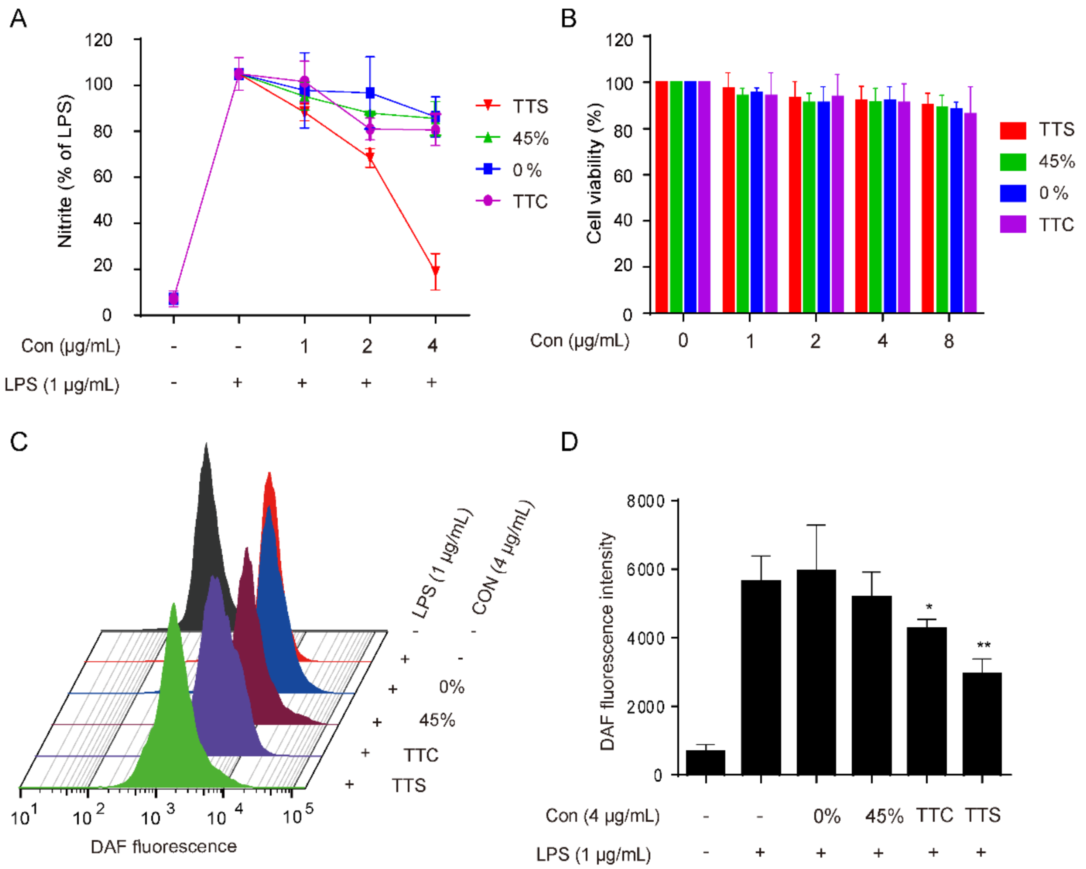

Figure 1S. The inhibitory nitric oxide (NO) release of TTS in LPS-stimulated RAW264.7 cells. (A). Cells were pretreated with 0% eluent, 45% eluent, TTC and TTS for 1 h before LPS (1μg/mL) co-culture for 24 h. The nitrite level of those sample were determined by Griess assay. (B) Cells were treated with 0% eluent, 45% eluent, TTC and TTS, respectively for 24 h. The cell viability was investigated by MTT assay. (C). Cells were individually treated with 0% eluent, 45% eluent, TTC and TTS for 6 h and then labeled with DAF-FM (1μM) for another 1 h. The NO release was determined by the flow cytometry. (D) The quantitative fluorescence intensity was statistically determined. $^{*}p< 0.05$ and $^{**}p< 0.01$versus LPS-treated group.

Table S1. Dynamic desorption capacities of two resins.

Resin	Desorption (mg)			
	Dihydrotanshinone	Tanshinone I	Cryptanshinone	Tanshinone IIA
D101	9.24±0.71	20.16±0.39	25.46±0.56	32.94±0.84
HPD100	8.83±0.54	19.87±0.73	25.41±0.38	31.43±1.34
